# Locally Aggressive Desmoid Tumor With L4–L5 Vertebral Invasion and Progressive Neurological Deficit: A Case Report

**DOI:** 10.1002/ccr3.73181

**Published:** 2026-07-16

**Authors:** Ghada A. AlAnsari, Ahmed Alanzi, Dawood Alatefi, Mohammed AlKhalaf, Sultan AlSalmi

**Affiliations:** ^1^ Department of Neurosurgery, King Fahad University Hospital Imam Abdulrahman Bin Faisal University Dammam Saudi Arabia; ^2^ Anaesthesia and Pain Management King Hamad University Hospital Al Sayh Bahrain; ^3^ Yemeni‐Syrian Medical Center Ja'ar Abyan Governorate Yemen; ^4^ Imam Abdulrahman Bin Faisal University Dammam Saudi Arabia

**Keywords:** desmoid tumor, desmoid‐type fibromatosis, lumbosacral spine, neurological deficit, spinal instability, vertebral invasion

## Abstract

Desmoid‐type fibromatosis is a rare, non‐metastatic fibroblastic neoplasm that may cause major morbidity through locally infiltrative growth. Spinal involvement with vertebral destruction and neurological deficit is particularly uncommon and may mimic malignant spindle‐cell or primary osseous tumors. We report a 59‐year‐old woman with severe low back pain, progressive right lower‐limb weakness, sensory loss, swelling, and functional decline. Imaging showed a markedly enlarging right retroperitoneal‐pelvic mass with invasion and partial destruction of the L4–L5 vertebral bodies. Although initially diagnosed as spindle cell sarcoma, histopathology, strong nuclear β‐catenin positivity, and CTNNB1 mutation analysis confirmed desmoid‐type fibromatosis. A pathogenic APC variant was also detected, raising the need to consider familial adenomatous polyposis evaluation. The patient underwent posterior decompression and long‐segment stabilization for neural compromise and spinal instability. This case highlights the severe local morbidity possible despite non‐metastatic histology.

## Key Clinical Message

Desmoid‐type fibromatosis should be considered in destructive lumbosacral spindle‐cell lesions, even when malignancy is initially suspected. Although non‐metastatic, it may cause severe local morbidity through vertebral invasion, neural encasement, spinal instability, and progressive neurological deficit. Early histopathologic confirmation and multidisciplinary management are essential.

## 1 Introduction

Desmoid‐type fibromatosis, also known as desmoid tumor or aggressive fibromatosis, is a rare intermediate fibroblastic neoplasm characterized by infiltrative local growth and absence of metastatic potential. Despite its non‐metastasizing behavior, it can cause substantial morbidity through progressive invasion of adjacent structures, pain, neurological compromise, and functional impairment. Current reviews and consensus recommendations emphasize that management should be guided not only by tumor size, but also by symptoms, anatomical site, growth behavior, and risk to critical structures [1, 2, 3, 4].

At the molecular level, most sporadic desmoid‐type fibromatoses are driven by activation of the Wnt/β‐catenin pathway, most commonly through CTNNB1 mutations. A smaller subset is associated with APC alterations and familial adenomatous polyposis, making recognition of APC findings clinically important [5, 6, 7]. Nuclear β‐catenin immunoreactivity is therefore an important diagnostic clue in the appropriate morphologic setting, although interpretation should be correlated with histopathologic findings, molecular testing, and differential diagnostic considerations [7, 8, 9, 10].

Desmoid‐type fibromatosis may arise in the abdominal wall, intra‐abdominally, or extra‐abdominally, with extra‐abdominal lesions more commonly involving the shoulder girdle, chest wall, trunk, or extremities [2, 3, 4]. In contrast, spinal and paraspinal involvement is distinctly uncommon, and vertebral invasion is particularly rare. Because of this unusual location, lumbosacral desmoid‐type fibromatosis may radiologically and clinically mimic malignant spindle‐cell or primary osseous tumors. Nevertheless, these lesions may produce severe neurological and functional consequences through local extension, vertebral destruction, spinal instability, and neural involvement [11, 12, 13, 14].

We report a rare case of locally aggressive desmoid‐type fibromatosis with L4–L5 vertebral invasion and progressive neurological deficit. This case highlights the diagnostic challenge, the relevance of molecular findings including CTNNB1 and APC alterations, and the disabling morbidity that may occur when a non‐metastatic tumor involves critical spinal structures.

## 2 Case History/Examination

A 59‐year‐old woman with a history of type 2 diabetes mellitus, dyslipidemia, and hyperthyroidism was admitted with severe low back pain, predominantly on the right side, radiating to the entire right lower limb, and associated with progressive weakness and numbness of the same limb. Her symptoms had started approximately 3 months earlier and progressively worsened. She had initially presented to another hospital, where she underwent CT‐guided biopsy of the lesion, which was reported as spindle cell sarcoma involving L5 with pelvic extension. The clinical course from symptom onset to diagnosis, intervention, and discharge is summarized in Table 1.

**TABLE 1:** Clinical timeline of presentation, diagnosis, treatment, and outcome.

Time point	Clinical event
Approximately 3 months before admission	Onset of severe right‐sided low back pain radiating to the right lower limb
Following weeks	Progressive worsening of pain, right lower‐limb weakness, numbness, inability to lie supine, and functional decline
Initial outside‐hospital evaluation	CT‐guided biopsy reportedly suggested spindle cell sarcoma involving L5 with pelvic extension
At presentation to our institution	Severe pain, right L4/L5 sensory deficit, marked right lower‐limb weakness, and right lower‐limb swelling
Diagnostic imaging	Large right retroperitoneal multilobulated mass with extrapelvic extension, complete invasion/partial destruction of L5, and partial invasion/destruction of L4
Histopathology and molecular testing	Spindle cell neoplasm with strong nuclear β‐catenin positivity; CTNNB1 p.(Ser45Pro) mutation and APC variant; no EWSR1 rearrangement or MDM2 amplification
Definitive diagnosis	Desmoid‐type fibromatosis with L4–L5 vertebral invasion
Surgical intervention	Posterior decompression and fixation from T12 to L5 with S1–S2 alar‐iliac screws
Postoperative course	Persistent severe pain, progressive right lower‐limb weakness, anemia, *Clostridioides difficile* infection, and candiduria
Discharge status	Hemodynamically stable but functionally dependent, with persistent neurological deficit and need for ongoing multidisciplinary management

The pain was severe, rated 10/10, and significantly affected her quality of life. It was aggravated by lying flat, rendering her unable to sleep in the supine position; instead, she slept sitting upright with pillows supporting her back. She denied urinary or fecal incontinence, headache, seizures, abnormal movements, visual disturbances, abdominal pain, chest pain, and fever. She reported unintentional weight loss of approximately 10 kg over the preceding 2 months.

Her past medical history was notable for intracranial hemorrhage 9 years earlier, for which she underwent angiography at another hospital, with a later MRI reportedly negative for bleeding. She also had acute right lower‐limb deep venous thrombosis, status post thrombectomy and inferior vena cava filter placement, as well as bilateral knee replacement 9 years earlier. Her sister had a history of breast cancer. She had no known drug or food allergies. Given the patient's history of right lower‐limb deep venous thrombosis and the presence of a large retroperitoneal‐pelvic mass, recurrent venous thrombosis and/or venous compression by the mass were considered possible contributors to the right lower‐limb swelling; however, the available records did not allow definitive attribution to either mechanism.

On examination, her vital signs were within normal limits. She was conscious, alert, and oriented, with a Glasgow Coma Scale score of 15/15, but appeared distressed because of severe pain. Cranial nerves were grossly intact. Motor examination showed full power in both upper limbs and in the left lower limb. In the right lower limb, there was marked weakness: hip flexion/extension 2/5, knee flexion/extension 3/5, ankle dorsiflexion 1/5, ankle plantarflexion 5/5, great toe plantarflexion 5/5, and great toe dorsiflexion 1/5. Reflexes were 2+ throughout. Sensory examination demonstrated reduced crude touch, light touch, and pinprick sensation in the right L4/L5 dermatomes. Plantar responses were downgoing bilaterally. Mild tenderness was present over the lumbosacral region. The right lower limb was swollen, with pitting edema extending to the knee and calf swelling. Abdominal examination was unremarkable.

## 3 Investigations and Treatment

Imaging demonstrated marked progression of a large right retroperitoneal multilobulated soft‐tissue mass with extrapelvic extension anterior to the right hip joint. The lesion measured 14 × 4 × 19 cm, compared with 6 × 5 × 4 cm on prior imaging. Although the exact interval between the two imaging studies was not fully documented in the available records, this change was interpreted as clinically significant enlargement during the symptomatic period. The mass extended into the lumbosacral region, completely invaded and partially destroyed the L5 vertebral body, and partially invaded and destroyed the right aspect of the L4 vertebral body. The destructive osseous involvement and mass effect correlated with the patient's severe lumbosacral pain, right L4/L5 sensory deficit, progressive right lower‐limb weakness, and mechanical instability (Figure 1).

**FIGURE 1:**
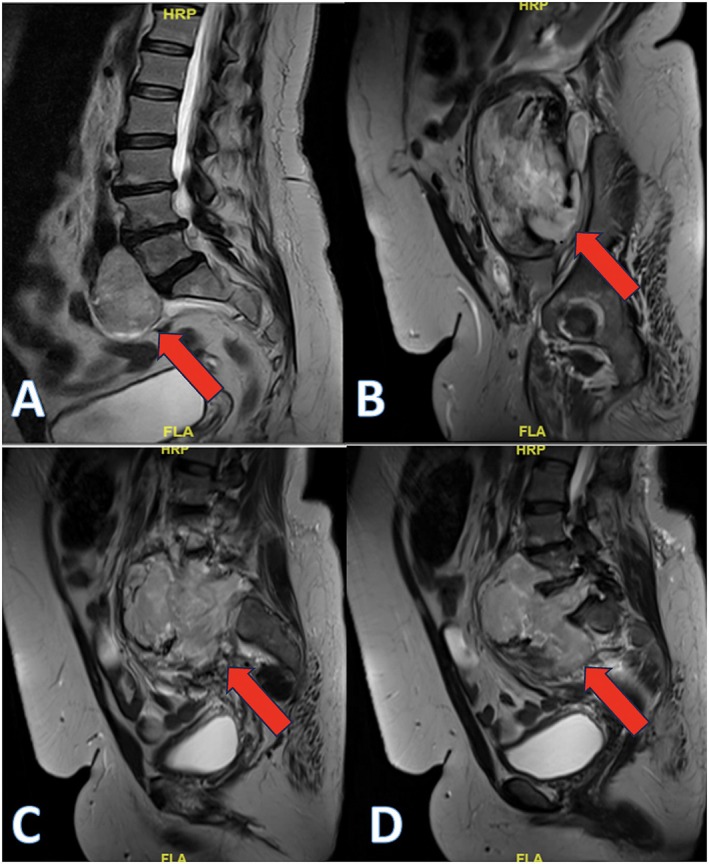
Magnetic resonance imaging of the lumbosacral spine and pelvis demonstrating locally aggressive desmoid‐type fibromatosis. (A) Sagittal lumbar MRI shows involvement of the lower lumbar spine with destructive changes centered around L4–L5/L5 and associated mass effect. (B) Pelvic MRI demonstrates a large right retroperitoneal multilobulated soft‐tissue mass extending toward the right pelvis/hip region. (C, D) Additional sagittal pelvic/lumbosacral images show the extent of the mass, with invasion and partial destruction of the L5 vertebral body and involvement of the right aspect of L4. The imaging findings explain the patient's severe lumbosacral pain, progressive right lower‐limb neurological deficit, and spinal instability.

Histopathological examination of the pelvic mass revealed a spindle cell neoplasm. Immunohistochemistry demonstrated strong nuclear β‐catenin positivity, with positivity for vimentin, caldesmon, cyclin D1, and focal smooth muscle actin, while desmin, S100, pan‐cytokeratin, DOG‐1, CD99, BCL2, estrogen receptor, and ALK‐1 were negative. CD34 showed only focal faint positivity. Molecular analysis identified a likely pathogenic CTNNB1 p.(Ser45Pro) mutation with a high variant allele frequency, supporting activation of the Wnt/β‐catenin pathway. A pathogenic APC variant was also detected. In addition, fluorescence in situ hybridization showed no evidence of EWSR1 rearrangement, and further testing demonstrated no MDM2 amplification. Taken together, these histopathological, immunophenotypic, and molecular findings confirmed the diagnosis of desmoid‐type fibromatosis (Figure 2).

**FIGURE 2:**
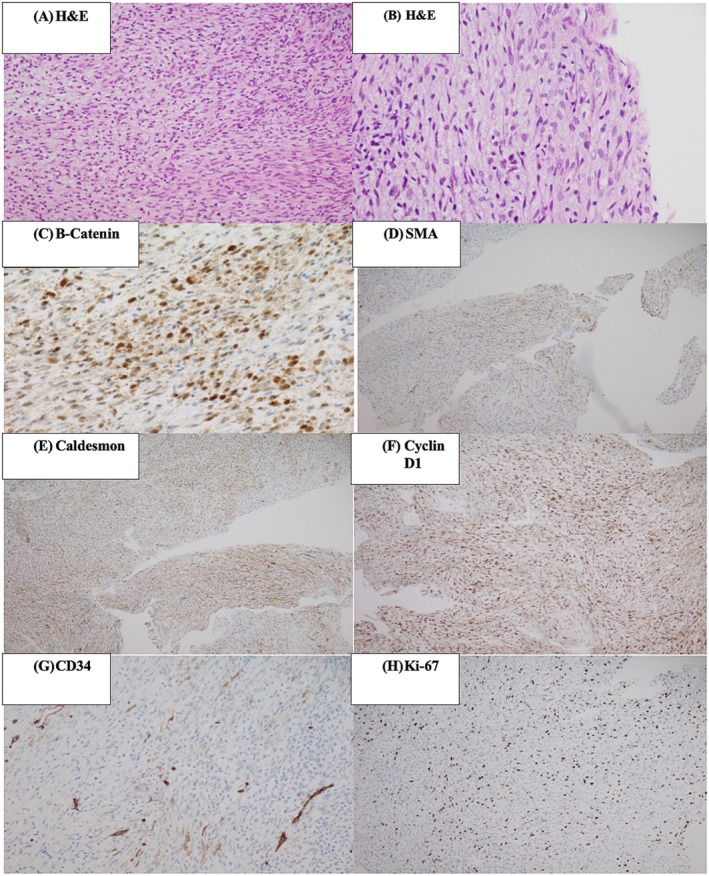
Histopathological and immunohistochemical findings of the pelvic mass. (A, B) Hematoxylin and eosin sections show a spindle‐cell neoplasm composed of bland fibroblastic/myofibroblastic cells arranged in sweeping fascicles within a collagenous stroma, without marked cytologic atypia. (C) Immunohistochemistry demonstrates strong nuclear β‐catenin expression, supporting desmoid‐type fibromatosis. (D–F) The tumor cells show immunoreactivity for smooth muscle actin, caldesmon, and cyclin D1. (G) CD34 staining is only focal/limited. (H) Ki‐67 shows a low proliferative index. The overall histomorphologic and immunohistochemical profile, together with CTNNB1 mutation analysis and absence of features supporting sarcoma or other spindle‐cell mimics, confirmed the diagnosis of desmoid‐type fibromatosis.

The detection of a pathogenic APC variant was considered clinically relevant because APC alterations may be associated with familial adenomatous polyposis‐related desmoid‐type fibromatosis. In the available clinical history, there was no documented personal history of colorectal polyposis or colorectal cancer. However, because formal familial adenomatous polyposis evaluation, colonoscopic screening, and genetic counseling were not fully documented in the available records, this remains an important limitation. Further genetic counseling and gastrointestinal evaluation were recommended to clarify whether the APC finding represented a germline alteration or a tumor‐associated molecular event.

The patient underwent posterior decompression and long‐segment stabilization from T12 to L5 with S1–S2 alar‐iliac screws using a CT‐guided navigation system. The surgical goal was to address neural compromise and mechanical instability rather than to achieve radical oncologic resection, because the lesion extensively involved the lumbosacral region and encased neural structures. Intraoperatively, the right L5 nerve root was found to be encased by the tumor. Given the destructive involvement of L4–L5/L5 and the need for spinopelvic stability, a long fixation construct with pelvic fixation was selected. Radical tumor debulking was not attempted because of the high risk of neurological injury and bleeding in the setting of extensive local disease.

Postoperatively, she was transferred to the intensive care unit and received 1 unit of packed red blood cells. She was later transferred to the regular ward; her hemoglobin had dropped to 6.3 g/dL, and she received 2 additional units of packed red blood cells, after which it improved to 8.9 g/dL. Computed tomography of the abdomen and pelvis with contrast, together with CT angiography, showed no evidence of active intralesional bleeding or contrast extravasation.

She underwent diagnostic spinal angiography with intersegmental artery embolization to reduce the risk of bleeding in preparation for a second‐stage surgical intervention. Her hospital course was further complicated by Clostridioides difficile infection, for which oral vancomycin was initiated, and candiduria, with urine culture positive for *Candida albicans* , for which antifungal therapy was started.

## 4 Outcome and Follow‐Up

At the most recent inpatient assessment, the patient remained hemodynamically stable and neurologically alert, with a Glasgow Coma Scale score of 15/15. The surgical wound was clean and dry, with no clinical signs of local wound infection. However, pain control remained challenging despite multimodal analgesia, including morphine, tramadol, gabapentin, paracetamol, and amitriptyline. She continued to experience severe breakthrough pain when the analgesic effect diminished.

Neurologically, the patient showed further deterioration of right lower‐limb power compared with the initial examination. Power was reduced to 1/5 in hip flexion and extension, 1/5 in knee flexion and extension, 1/5 in ankle dorsiflexion, 4/5 in ankle plantarflexion, 4/5 in great toe plantarflexion, and 1/5 in great toe dorsiflexion. Right lower‐limb swelling also increased during admission. Given the patient's previous history of right lower‐limb deep venous thrombosis and the presence of a large retroperitoneal‐pelvic mass, recurrent venous thrombosis and/or venous compression were considered possible contributors; however, the available records did not allow definitive attribution of the worsening swelling to either mechanism.

The preoperative and most recent inpatient/postoperative neurological findings are summarized in Table 2.

**TABLE 2:** Comparison of preoperative and postoperative neurological findings.

Neurological parameter	Preoperative examination	Most recent inpatient/postoperative assessment
Mental status	Conscious, alert, and oriented; GCS 15/15	Conscious and alert; GCS 15/15
Upper limbs	Full power bilaterally	No new upper‐limb deficit reported
Left lower limb	Full power	No left lower‐limb deterioration reported
Right hip flexion/extension	2/5	1/5
Right knee flexion/extension	3/5	1/5
Right ankle dorsiflexion	1/5	1/5
Right ankle plantarflexion	5/5	4/5
Right great toe dorsiflexion	1/5	1/5
Right great toe plantarflexion	5/5	4/5
Sensory examination	Reduced crude touch, light touch, and pinprick sensation in the right L4/L5 dermatomes	Persistent neurological deficit
Reflexes	2+ throughout	Not specifically changed
Plantar response	Downgoing bilaterally	Not specifically changed
Functional status	Severe pain with functional decline	Functionally dependent at discharge

Given the extensive local disease, neural encasement, vertebral destruction, incomplete functional recovery, and persistent severe pain, the patient required ongoing multidisciplinary care involving spine surgery, oncology, pain management, rehabilitation, and follow‐up imaging. She was eventually discharged to another facility for continued care but remained functionally dependent at discharge.

Long‐term follow‐up after transfer was limited. Therefore, later oncologic treatment, response to therapy, neurological recovery, radiologic progression, recurrence status, and long‐term functional outcome could not be fully assessed. This represents an important limitation of the present report, particularly because desmoid‐type fibromatosis may follow a variable clinical course and requires longitudinal assessment to evaluate stability, progression, or treatment response.

## 5 Discussion

### 5.1 Locally Aggressive Behavior Despite Non‐Metastatic Biology

Desmoid‐type fibromatosis is a rare fibroblastic neoplasm characterized by infiltrative local growth and absence of metastatic potential. Although histologically non‐metastatic, it may cause substantial morbidity when it arises near critical neurovascular, visceral, or skeletal structures. Contemporary reviews and consensus recommendations emphasize that management should be guided by tumor location, symptoms, progression, and risk to function, rather than histology alone [1, 2, 3, 4]. This principle is illustrated in the present case, in which a retroperitoneal‐pelvic desmoid‐type fibromatosis caused L4–L5 vertebral invasion, severe refractory pain, progressive neurological deficit, spinal instability, and major functional decline.

### 5.2 Diagnostic Challenge, Molecular Findings, and APC/FAP Relevance

Most sporadic desmoid‐type fibromatoses are associated with activation of the Wnt/β‐catenin pathway, commonly through CTNNB1 mutations, whereas a smaller subset is associated with APC alterations and familial adenomatous polyposis [5, 6, 7]. Nuclear β‐catenin immunoreactivity is therefore an important diagnostic clue when interpreted in the appropriate morphologic and clinicoradiologic context [7, 10]. In this case, the initial outside‐hospital diagnosis of spindle cell sarcoma highlights the diagnostic difficulty posed by destructive spinal and retroperitoneal spindle‐cell lesions.

The differential diagnosis included spindle cell sarcoma, fibrosarcoma, leiomyosarcoma, malignant peripheral nerve sheath tumor, gastrointestinal stromal tumor, solitary fibrous tumor, inflammatory myofibroblastic tumor, and primary osseous tumors involving the lumbar spine. Desmoid‐type fibromatosis was supported by bland spindle‐cell morphology, strong nuclear β‐catenin positivity, and identification of a CTNNB1 mutation [7, 8, 9, 10]. Alternative diagnoses were considered less likely because S100 negativity argued against neural differentiation, pan‐cytokeratin negativity argued against epithelial malignancy, DOG‐1 negativity argued against gastrointestinal stromal tumor, ALK‐1 negativity argued against inflammatory myofibroblastic tumor, and absence of EWSR1 rearrangement and MDM2 amplification helped exclude selected sarcoma entities and dedifferentiated liposarcoma. These findings underscore the importance of integrating histology, immunohistochemistry, molecular testing, and imaging before establishing the final diagnosis.

The APC variant identified in this case is clinically important because desmoid‐type fibromatosis may occur either sporadically, most often in association with CTNNB1 mutations, or in the setting of APC alterations and familial adenomatous polyposis. Although the CTNNB1 p.(Ser45Pro) mutation strongly supported the diagnosis of desmoid‐type fibromatosis, the concurrent APC finding raised the question of an underlying hereditary predisposition. In the absence of documented colonoscopic evaluation or formal genetic counseling, familial adenomatous polyposis could not be definitively excluded. This should be acknowledged as a limitation, and such patients should be considered for genetic counseling and gastrointestinal evaluation when clinically appropriate.

### 5.3 Rarity and Significance of Lumbosacral Involvement

Extra‐abdominal desmoid‐type fibromatosis more commonly involves the trunk, shoulder girdle, chest wall, or extremities, whereas spinal and paraspinal involvement is distinctly uncommon [2, 3, 4]. Vertebral invasion is particularly rare and has mainly been described in isolated case reports and small series [11, 12, 13, 14]. Previously reported spinal or paraspinal cases have included lumbar facet joint involvement, de novo spinal fibromatosis, and postoperative fibromatosis after spinal instrumentation [11, 12, 13, 14]. Some lesions were primarily paraspinal or postoperative, while others involved posterior spinal elements or developed in relation to previous spinal procedures.

Compared with these reports, the present case is notable for a large retroperitoneal‐pelvic mass with destructive L4–L5/L5 vertebral invasion, encasement of the right L5 nerve root, progressive motor and sensory deficit, spinal instability requiring long‐segment fixation with pelvic fixation, and severe functional decline. This combination of extensive vertebral destruction, neural encasement, and progressive neurological deterioration emphasizes the unusual morbidity that may occur despite the non‐metastatic biology of desmoid‐type fibromatosis.

### 5.4 Management Considerations in High‐Risk Spinal Disease

Management of desmoid‐type fibromatosis has shifted toward active surveillance for selected asymptomatic or mildly symptomatic patients, as some lesions may stabilize or regress spontaneously [1, 3, 15]. However, active surveillance is not appropriate when disease is progressive, painful, function‐threatening, or anatomically hazardous [1, 3, 4, 15]. In our patient, severe pain, progressive motor and sensory deficits, L4–L5 vertebral destruction, neural encasement, and mechanical instability justified surgical intervention. In this context, the surgical goal was decompression and stabilization rather than radical oncologic resection, and radical tumor debulking was not attempted because of the high risk of neurological injury and bleeding. Posterior decompression and long‐segment stabilization from T12 to L5 with S1–S2 alar‐iliac screws were therefore performed to address neural compromise and spinopelvic instability.

The surgical goal was decompression and stabilization rather than radical oncologic resection. This distinction is important because complete resection may be unsafe or unachievable when desmoid‐type fibromatosis encases neural structures, invades bone, or extends into adjacent soft tissues. In the present case, posterior decompression and long‐segment stabilization from T12 to L5 with S1–S2 alar‐iliac screws were performed to address neural compromise and spinopelvic instability. The use of a long fixation construct with pelvic fixation was justified by the destructive involvement of L4–L5/L5 and the need for durable lumbopelvic stability. Radical tumor debulking was not documented in the available operative details, likely reflecting the high risk of neurological injury and bleeding in the setting of extensive local disease.

### 5.5 Outcome, Follow‐Up, and Therapeutic Implications

This case also demonstrates the limitations of surgery in advanced desmoid‐type fibromatosis involving critical spinal anatomy. Despite decompression and fixation, the patient continued to experience severe pain and progressive right lower‐limb weakness and remained functionally dependent at discharge. Ongoing management in such cases requires coordination among spine surgery, oncology, radiology, pain medicine, and rehabilitation. For progressive, unresectable, or morbid desmoid‐type fibromatosis, systemic therapy may be considered, and recent phase 3 evidence has shown clinical benefit with nirogacestat in adults with progressing desmoid tumors [16].

The main limitation of this case report is the absence of meaningful long‐term follow‐up after discharge to another facility. As a result, we could not assess later neurological recovery, radiologic progression, recurrence, response to systemic therapy or radiotherapy, or long‐term functional outcome. Nevertheless, the case remains clinically educational because it documents a rare presentation of desmoid‐type fibromatosis with destructive lumbosacral vertebral invasion, neural encasement, progressive neurological deficit, spinal instability, and major functional decline.

In conclusion, desmoid‐type fibromatosis should be considered in the differential diagnosis of destructive lumbosacral spindle‐cell lesions, even when malignancy is initially suspected. Although non‐metastatic, it may behave aggressively in high‐risk anatomical sites and cause severe local morbidity. Early diagnosis, accurate histopathologic and molecular confirmation, assessment for possible hereditary predisposition when APC alterations are detected, and timely multidisciplinary management are essential.

## Author Contributions

**Dawood Alatefi:** conceptualization, writing – original draft, investigation, methodology, validation, visualization, writing – review and editing, resources, supervision, formal analysis. **Ahmed Alanzi:** conceptualization, investigation, writing – original draft, methodology, validation, visualization, writing – review and editing, software, data curation. **Ghada A. AlAnsari:** conceptualization, investigation, writing – original draft, methodology, validation, visualization, writing – review and editing, software, supervision, data curation. **Mohammed AlKhalaf:** writing – original draft, methodology, formal analysis, data curation. **Sultan AlSalmi:** supervision, writing – review and editing, methodology, conceptualization.

## Funding

The authors have nothing to report.

## Consent

Written informed consent was obtained from the patient for publication of this case report and any accompanying images. The consent form is available for review by the Editor‐in‐Chief upon request.

## Conflicts of Interest

The authors declare no conflicts of interest.

## Data Availability

The data that support the findings of this study are available from the corresponding author upon reasonable request.
